# Proper symmetric and asymmetric endoplasmic reticulum partitioning requires astral microtubules

**DOI:** 10.1098/rsob.150067

**Published:** 2015-08-19

**Authors:** Jeremy T. Smyth, Todd A. Schoborg, Zane J. Bergman, Blake Riggs, Nasser M. Rusan

**Affiliations:** 1 Cell Biology and Physiology Center, National Heart, Lung, and Blood Institute, National Institutes of Health, Bethesda, MD, USA; 2 Department of Anatomy, Physiology, and Genetics, Uniformed Services University of the Health Sciences-F. Edward Hébert School of Medicine, Bethesda, MD 20814, USA; 3 Department of Biology, San Francisco State University, 1600 Holloway Avenue, San Francisco, CA 94132, USA

**Keywords:** endoplasmic reticulum, centrosome, asymmetric division, *Drosophila*, mitosis

## Abstract

Mechanisms that regulate partitioning of the endoplasmic reticulum (ER) during cell division are largely unknown. Previous studies have mostly addressed ER partitioning in cultured cells, which may not recapitulate physiological processes that are critical in developing, intact tissues. We have addressed this by analysing ER partitioning in asymmetrically dividing stem cells, in which precise segregation of cellular components is essential for proper development and tissue architecture. We show that in *Drosophila* neural stem cells, called neuroblasts, the ER asymmetrically partitioned to centrosomes early in mitosis. This correlated closely with the asymmetric nucleation of astral microtubules (MTs) by centrosomes, suggesting that astral MT association may be required for ER partitioning by centrosomes. Consistent with this, the ER also associated with astral MTs in meiotic *Drosophila* spermatocytes and during syncytial embryonic divisions. Disruption of centrosomes in each of these cell types led to improper ER partitioning, demonstrating the critical role for centrosomes and associated astral MTs in this process. Importantly, we show that the ER also associated with astral MTs in cultured human cells, suggesting that this centrosome/astral MT-based partitioning mechanism is conserved across animal species.

## 1. Introduction

Cells face a significant challenge each time they divide, because not only must they faithfully replicate and partition their genomic DNA, they must also expand and partition their cytoplasmic contents and organelles as well. It is particularly important that cells inherit sufficient quantities of functionally competent organelles with each division as cells cannot generate many of their organelles de novo [1]. Despite this, mechanisms that ensure proper partitioning of organelles during cell division, particularly membrane-bound organelles like mitochondria and the endoplasmic reticulum (ER), are poorly understood. Delineating these mechanisms is key to understanding how organelle-specific functions regulate proper development, tissue homeostasis and injury repair [2].

The ER is the largest membrane-bound organelle in the cell, and its functions include folding and trafficking of secretory proteins, lipid synthesis and transport, and regulation of cytoplasmic Ca^2+^. During interphase, the ER is continuous with the nuclear envelope (NE) and is distributed throughout the cytoplasm as a network of broad sheets, or cisternae, and thin tubules [3]. This interphase ER distribution depends in large part on numerous associations with the microtubule (MT) cytoskeleton, which involve MT motor-dependent transport, connections with growing MT tips and stable attachments along MT filaments [4]. Importantly, the roles of particular ER morphologies and MT associations in specific ER and cellular functions are poorly understood. It is also unclear whether specific regulation of ER morphogenesis or distribution is required during cell division for the proper execution of mitosis or to ensure functional ER partitioning to progeny cells.

Two hypotheses have been proposed to explain ER partitioning and inheritance during cell division [2]. The first proposes that the ER is actively segregated during division, probably through interactions with cytoskeletal elements. This would provide a mechanism for specific regulation of ER partitioning to progeny cells. In support of this, and consistent with the association of the ER with MTs during interphase, the ER localizes to the MT-based mitotic spindle in a variety of cell types from different species including sea urchin [5] and *Drosophila* [6] embryos and mammalian tissue culture cells [7]. Thus, it is expected that disruption of ER–spindle interactions would disrupt ER functions in progeny cells. However, the specific factors that physically link the ER with spindle MTs have not been identified in any animal cell type, and this has precluded a direct test of whether the ER–spindle association is required for functional ER partitioning. Further, several recent studies showing that the ER remains mostly peripheral to the mitotic spindle with no obvious MT contacts, particularly in cultured human cells [8,9], have challenged the idea that spindle association is a universal requirement for ER partitioning. These findings support the second hypothesis, which proposes that stochastic distribution of the ER throughout a dividing cell is sufficient to ensure adequate partitioning to progeny cells. Thus, although the ER is associated with MTs in some dividing cells, this active segregation may not be strictly required as long as each progeny cell acquires enough organelle material. However, it is notable that dissociation of the ER from spindle MTs is most readily apparent in cultured cells such as HeLa and Cos-7, and these cells may not have strict requirements for precise ER inheritance. By contrast, when cells divide in the context of a developing organism in which spatial and temporal coordination of cellular events is crucial, small alterations to ER partitioning may have far-reaching effects. This illustrates the critical importance of studying mitotic ER partitioning in cells dividing within intact, developing tissues, in order to understand how the partitioning mechanisms function within physiological cellular processes.

A striking example of how active segregation of cellular components during cell division can have significant consequences for progeny cells within a developing or functional tissue is asymmetric stem cell division. During asymmetric stem cell division, differential partitioning of specific factors results in two progeny cells with different identities or fates, most commonly with one cell programmed to remain a stem cell and the second cell becoming a tissue-specific effector [10]. The establishment of asymmetry in these dividing cells raises an important question that has never been addressed: is the ER asymmetrically partitioned during asymmetric stem cell division? If so, then this would strongly support the hypothesis that highly regulated, active segregation of the ER is required during *in vivo* cell division. Further, by integrating ER dynamics with known mechanisms that establish asymmetry in these cells, we may be able to glean novel insights into ER partitioning mechanisms. We have taken this approach in the current study by analysing ER partitioning in asymmetrically dividing *Drosophila* neural stem cells known as neuroblasts (NBs). Asymmetric NB divisions produce a large cell that retains NB identity, and a much smaller ganglion mother cell (GMC) that differentiates to form a functional neuron or glial cell [11]. Our analyses define an asymmetric segregation of the ER to the mitotic spindle poles that results in a larger proportion of the organelle being partitioned to the future stem cell. We also show that active, MT-dependent spindle pole segregation is required *in vivo* for proper ER partitioning in both asymmetrically and symmetrically dividing cells, as well as in human culture cells. Thus, active spindle pole segregation may be a highly conserved mechanism of ER partitioning that can be subject to precise regulation during specific developmental processes, such as asymmetric stem cell division.

## 2. Material and methods

### 2.1. Fly stocks

The following Flytrap lines were used in this study: CC00735 (GFP-Sec61*α*), G00199 (GFP-Rtnl-1) [12] and ZCL1514 (GFP-PDI) [13]. H2A-mRFP and the *asp* mutant were obtained from the Bloomington *Drosophila* Stock Center (stock numbers 23651 and 29202, respectively); *asl^MecD^* was a gift from Dr Tomer Avidor-Reiss.

### 2.2. Live imaging of *Drosophila* tissues

Whole brains or testes were dissected from third-instar larvae in *Drosophila* Schneider's medium (Life Technologies) containing Antibiotic-Antomycotic (Life Technologies) and were mounted in the same medium for imaging on a 50 mm gas-permeable lumox dish (Sarstedt). The medium was surrounded by Halocarbon 700 oil (Sigma) to support a glass coverslip (22 × 22 mm, #1.5, Fisher) that was placed on top of the medium [14]. The dish was flipped and placed in a stage incubator heated to 25°C (Bionomic System BC-110, 20/20 Technologies) on the stage of an inverted microscope (Eclipse Ti, Nikon) equipped with a spinning disc confocal head (CSU-22, Yokogawa) and a cooled charge-coupled device camera (Flash4, Hamamatsu). Filters were controlled by an automated controller (MAC 6000, Ludl), and excitation light was provided by 491, 561 and 642 nm solid-state lasers housed in a single laser merge module (VisiTech International). All components were run by MetaMorph software (Molecular Devices). Fifteen images at 1 µm intervals were captured every 2 min. Images were processed and videos were compiled using ImageJ software (NIH). For embryo imaging, 20–30 mated female flies were housed at 25°C for 1 h on grape-juice agar plates with a smear of active yeast paste covered by a perforated beaker. Embryos were collected from the plate and washed three times in PBS, de-chorionated for 5 min in 100% bleach and washed an additional three times in PBS. A drop of embryos in PBS was placed on a glass coverslip, excess PBS was removed and the embryos were covered in a drop of Aqua-Poly/Mount mounting medium (Polysciences). A lumox dish was then placed on top of the mounting medium and rotated to disperse the medium and embryos. The mounted embryos were imaged at 25°C by spinning disc confocal microscopy as described above.

### 2.3. Embryo microinjection

Embryos were collected on grape-juice agar plates, aged on collection plates and dechorionated by hand. Dechorionated embryos were briefly desiccated and microinjected as previously described [15]. The needle concentrations of rhodamine-labelled tubulin (Cytoskeleton Inc.) were 2 mg ml^−1^. Confocal images of injected embryos were obtained with a Zeiss Cell Observer instrument (Carl Zeiss Microimaging, Inc.) using the 488 nm and 543 nm wavelengths from an argon laser and a C-Apochromat 1.2 NA 100× objective. Images were analysed with ImageJ and Axiovision (Carl Zeiss MicroImaging, Inc).

### 2.4. Culture and live imaging of S2 and HeLa cells

S2 cells (*Drosophila* Genomics Resource Center) were grown in *Drosophila* Schneider's medium supplemented with 10% fetal bovine serum (Life Technologies) and Antibiotic-Antimycotic at room temperature in air. For live imaging, cells were plated for 1 h in supplemented Schneider's medium in glass-bottom dishes (MatTek) coated with concanavalin A (0.5 mg ml^−1^, Sigma). Cells were imaged at 25°C by spinning disc confocal microscopy as described above. HeLa cells (ATCC) were grown in DMEM (Life Technologies) supplemented with 10% fetal bovine serum at 37°C in 5% CO_2_. Cells were plated in glass-bottom dishes overnight for live spinning disc confocal imaging. Prior to imaging, culture medium was replaced with Leibovitz's L-15 medium (Life Technologies) supplemented with 10% fetal bovine serum, and cells were imaged at 37°C in air.

### 2.5. Fixation and immunofluorescence

Whole third-instar larval *Drosophila* brains and testes were fixed for 20 min at room temperature in PBST (PBS with 0.3% Triton-X 100) containing 8% paraformaldehyde (Electron Microscopy Sciences). Fixed tissues were then washed three times in PBST, incubated overnight at 4°C with rotation in primary antibodies diluted in PBST with 5% bovine serum albumin (BSA), washed three times in PBST, incubated 4 h at room temperature in secondary antibodies in PBST with 5% BSA, washed three times in PBST and mounted in Auqa-Poly/Mount mounting medium. S2 cells were prepared for immunofluorescence by plating them in Schneider's medium on concanavalin A coated glass coverslips for 1 h at room temperature. Cells were then fixed in 100% methanol at −20°C for 20 min and washed three times in PBS. HeLa cells for immunofluorescence were grown on glass coverslips overnight and fixed as described [16]. Fixed HeLa and S2 cells were blocked for 1 h at room temperature in PBT (PBS with 0.1% Tween-20), incubated in primary antibody diluted in PBT with 1% BSA for 1 h at room temperature, washed three times in PBT, incubated in secondary antibody diluted in PBT with 1% BSA, washed three times and mounted in Auqa-Poly/Mount mounting medium. Primary antibodies used were mouse anti-*α*-tubulin (DM1*α*, 1 : 200, Sigma), anti-phosphorylated histone H3 (1 : 1000, EMD Millipore) guinea pig anti-asl (1 : 10 000, gift from G. Rogers, University of Arizona Cancer Center) and anti-baz (1 : 2000, gift from T. Harris, University of Toronto). Secondary antibodies were Alexa Fluor 488, 568 or 647 (Life Technologies) at 1 : 500.

### 2.6. Plasmids and transfections

The coding sequence of *Drosophila Sec61α* was cloned by PCR from EST LD29847 from the DGRC Gold Collection and inserted into the pENTR/D-TOPO plasmid (Life Technologies) according to manufacturer's instructions. This was then recombined with Gateway Destination Vectors that placed EGFP or tagRFP at the C-terminus of *Sec61α* under control of the *Drosophila ubiquitin* promoter sequence. S2 cells were transfected with 2 µg plasmid using Effectene (Qiagen) or Amaxa nucleofection according to manufacturers' instructions. For combined expression of RFP-Sec61*α* and GFP-*α*-tubulin, an S2 line stably expressing GFP-*α*-tubulin (DGRC) was transfected with RFP-Sec61*α*. Plasmid encoding EGFP-tagged human Sec61*β* was obtained from Addgene and transfected into HeLa cells with Lipofectamine 2000 according to manufacturer's instructions.

### 2.7. Quantification of endoplasmic reticulum asymmetry

GFP-Sec61*α* expressing NBs were imaged live, and Z-projections that encompassed the entire ER network were created for each cell at metaphase. The total fluorescence within equally sized regions (approx. 2.25 µm in diameter) centred around each spindle pole was calculated using the Integrated Density function of ImageJ. Background from identical regions was subtracted from each pole measurement to obtain corrected ER fluorescence intensities at each apical and basal pole. Total cellular ER fluorescence intensities were similarly calculated from the same images using regions that encompassed all of the ER. Each apical and basal measurement was then expressed as a percentage of the total ER measurement for each cell. Data are presented as mean ± s.e.m. and statistical significance was calculated using the Student's *t*-test.

## 3. Results

### 3.1. Spindle poles asymmetrically partition the endoplasmic reticulum in dividing *Drosophila* neuroblasts

**Figure 1.**
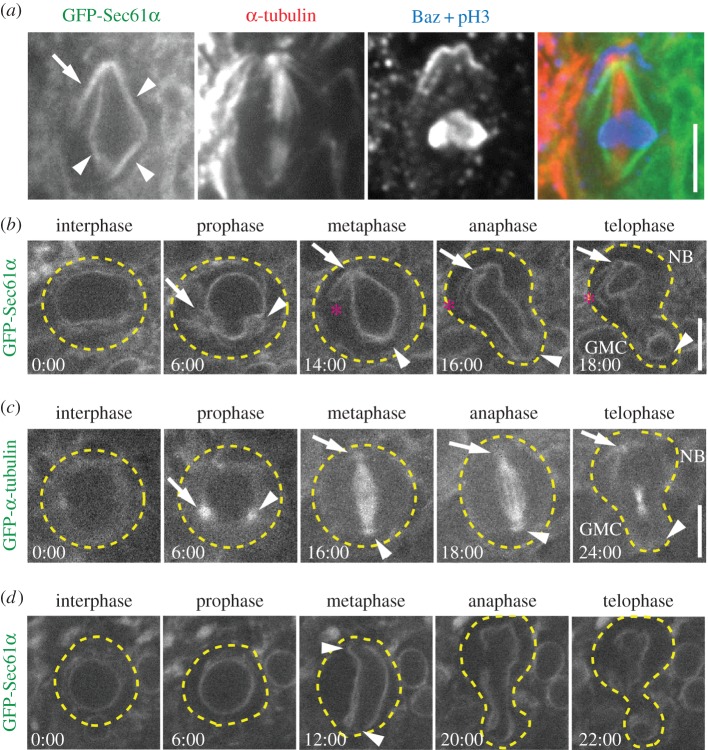
The ER is asymmetrically partitioned in dividing NBs. (*a*) A fixed metaphase NB expressing GFP-Sec61*α* (green) was immunostained for *α*-tubulin (red), Baz (blue) and phosphorylated histone 3 (pH3, blue). Indicated are the ER envelope (arrowheads) and the apical ER extension (arrow). (*b*) A single GFP-Sec61*α* expressing NB was imaged live throughout the course of a single mitotic division. Depicted are the approximate outline of the cell (dotted lines), apical centrosome (arrow), the basal centrosome (arrowhead) and the apical ER extension (asterisk). (*c*) A single GFP-*α*-tubulin expressing NB was imaged live throughout the course of a single mitotic division. The apical centrosome (arrow) and the basal centrosome (arrowhead) are shown. (*d*) An *asl^mecD^*/*asl^mecD^*, GFP-Sec61*α* expressing NB was imaged live throughout the course of a single mitotic division. Indicated are the approximate outline of the cell (dotted lines) and the spindle poles (arrow heads). Note the lack of centrosomal and spindle pole accumulations of ER. Times, min:s; scale bar, 5 µm.

Asymmetrically dividing stem cells provide a novel and powerful physiological system in which to investigate the cellular mechanisms that regulate ER partitioning during cell division. To carry out this analysis, we first analysed metaphase third-instar larval *Drosophila* NBs to determine whether there are specific differences in ER localization or distribution along the cell's apico-basal axis. Green fluorescent protein (GFP)-tagged Sec61*α* was used to label ER membranes relative to apical and basal domains of mitotic NBs as defined by immunostaining for the *Drosophila* PAR3 orthologue Bazooka (Baz). We found that the ER was predominantly organized as an envelope immediately surrounding the mitotic spindle MTs (figure 1*a*, arrowheads); we will refer to this structure as the ‘ER envelope’. Consistently, we also identified an extension of ER membrane outside of the ER envelope, specifically positioned near the apical spindle pole (figure 1*a*, arrow). An analogous ER extension was not seen at the opposite, basal pole. This was a surprising result that led to the hypothesis that asymmetrically dividing NBs organize and distribute ER asymmetrically to the two daughter cells at each division. To test this hypothesis, we carried out live *in vivo* imaging of NBs [14] with the goal of determining when ER asymmetry is established in the cell cycle and how the ER is distributed to the progeny NB versus GMC. We determined cell-cycle stage based on the shape of the ER envelope and by timing relative to anaphase onset, while apico-basal polarity was determined based on the size of the progeny cells following division. We found that the ER is uniformly distributed throughout the cytoplasm and around the nucleus during interphase (figure 1*b*; electronic supplementary material, video S1), though the small size of NBs and dim fluorescence prevented us from differentiating between sheet and tubular ER. During prophase, the NE became spherical with the exception of two conspicuous indentations, probably formed by the two centrosomes as they migrated around the NE to opposite poles (figure 1*b*, arrow and arrowhead). As the amount of ER increased around the nucleus and the centrosomes, there was a concomitant decrease in cytoplasmic ER. This observation is consistent with the presence of an active mechanism that rearranges the ER during mitotic entry, similar to the tubule to sheet transformation documented in mammalian cultured cells [9]. Notably, ER was recruited to the centrosomes early in mitosis, suggesting that centrosomes are key regulators of ER redistribution. Remarkably, one of the centrosomes (figure 1*b*, arrow) was associated with significantly more ER than the other (figure 1*b*, arrowhead). Furthermore, tracking the two centrosomes and associated ER as they fully separated to opposite sides of the nucleus allowed us to determine that the centrosome with more associated ER formed the apical spindle pole, destined for the NB. Similar analysis of GFP-*α*-tubulin showed that the centrosome that formed the apical spindle pole also had a higher density of MTs during prophase (figure 1*c*, arrow; electronic supplementary material, video S2), consistent with previously shown centrosome asymmetry at this stage [17,18]. This relationship between ER and MT asymmetry at the two centrosomes suggests that centrosomal MTs may establish ER asymmetry early in mitosis (prophase prior to NEB)) in NBs. Once spindle assembly was complete in metaphase, a prominent ER extension from the apical, but not the basal, spindle pole was detected (figure 1*b*, asterisk, *n* = 17/20), similar to the ER extension observed in fixed cells (figure 1*a*). Based on fluorescence intensity quantification during metaphase, there is 20% more ER located at the apical versus the basal spindle pole (12.22 ± 2.63% versus 10.16 ± 2.31% of the total cellular ER, respectively), a statistically significant difference (*n* = 16 cells; *p* < 0.01). Importantly, as the cell exited mitosis, the entire apical ER domain specifically partitioned to the regenerating NB and not the differentiating GMC. Although the volume of the NB is much greater than the GMC and would naturally sequester more ER following division, the additional apical ER extension we identified could effectively result in a higher ER concentration in the NB versus GMC. Collectively, we show that the ER is recruited to centrosomes early in mitosis with more organelle material organized by the apical centrosome and inherited by the neural stem cell, potentially resulting in a higher ER concentration needed for NB function.

Our results suggest that centrosomes do not merely correlate with the position of ER membranes, but are critical regulators of this ER asymmetry. To determine whether centrosomes are in fact required for establishing ER asymmetry, we analysed NBs from animals lacking centrosomes due to a mutation in the *asterless* (*asl*) gene. Although *asl* mutant cells can form spindles and divide, they lack astral MTs [19]. NBs from animals homozygous for the loss of function *asl^mecD^* allele show normal perinuclear ER distribution (figure 1*d*; electronic supplementary material, video S3), but lack centrosomal/polar accumulation of ER, as expected. Importantly, during metaphase when the ER envelope adopted the typical diamond shape, no ER extensions or additional accumulations at either spindle pole were detected (figure 1*d*, arrows). This cell still established an asymmetrically located cleavage furrow, typical of about 95% of *asl* mutant NBs [17,18], and ER membranes are segregated to both the NB and GMC. However, the NB did not inherit any additional apical spindle pole-dependent ER seen in wild-type cells, suggesting that the concentration of ER in the NB and GMC are equalized. We conclude that spindle poles organized by functional centrosomes are required for asymmetric ER partitioning that is established early in mitosis in NBs, which could lead to higher NB ER concentration that could be physiologically relevant.

### 3.2. Spindle pole-organized microtubules are required for proper endoplasmic reticulum partitioning

**Figure 2.**
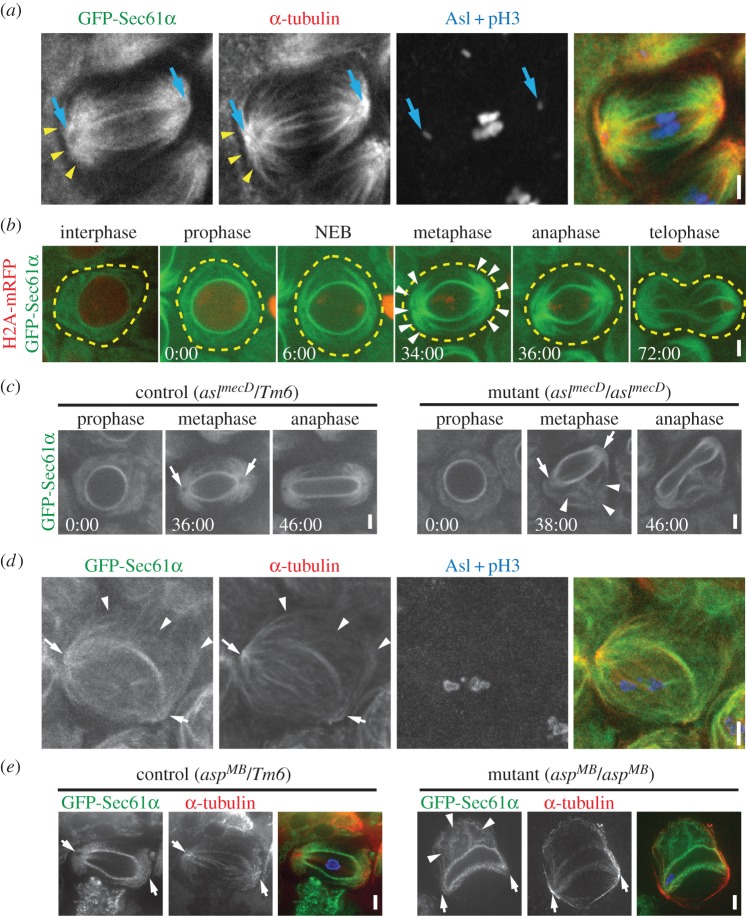
The ER associates with astral MTs in meiotic spermatocytes. (*a*) A fixed GFP-Sec61*α* (green) spermatocyte at metaphase of the first meiotic division was immunostained for *α*-tubulin (red), asterless (Asl, blue) to localize centrosomes (blue arrows) and phosphorylated histone 3 (pH3, blue). ER structures are closely aligned with astral MTs (yellow arrowheads). (*b*) A spermatocyte expressing GFP-Sec61*α* (green) and H2A-mRFP (red) was imaged live throughout the course of the first meiotic division. NEB was determined based on the sudden loss of background H2A-mRFP fluorescence throughout the nucleus. The approximate outline of the cell (yellow dotted lines) and the astral ER domains at metaphase (arrow heads) are indicated. (*c*) *asl^mecD^*/*TM6* control (left panel) and *asl^mecD^*/*asl^mecD^* (right panel) GFP-Sec61*α* expressing spermatocytes were imaged throughout meiosis I. Spindle poles (arrows) recruit ER in control cells, but fail to do so in *asl^mecD^*/*asl^mecD^* spermatocyte; abnormal ER structures (arrowheads) are prominent in the *asl^MecD^*/*asl^MecD^* spermatocyte. (*d*) A fixed GFP-Sec61*α* (green) metaphase I *asl^MecD^*/*asl^MecD^* spermatocyte was immunostained for *α*-tubulin (red), asl (blue) and pH3 (blue). Indicated are spindle poles (arrows) and abnormal ER structures associated with MTs (arrowheads). (*e*) Fixed GFP-Sec61*α* (green) metaphase I *asp^MB^*/*TM6* control (left panel) and *asp^MB^*/*asp^MB^* (right panel) spermatocytes were fixed and immunostained for *α*-tubulin (red). DAPI staining of DNA is shown in blue in the merged image. Abnormal ER structures are prominent in *asp^MB^*/*asp^MB^*spermatocyte (arrows). Times, min:s; scale bar, 5 µm.

Based on our data, one possibility is that NBs have adapted or modified a universal spindle pole-dependent ER partitioning mechanism to achieve functional asymmetry of the organelle. This prompted us to investigate the spindle pole-dependence of the ER in other *Drosophila* cell types. We began by analysing meiotic spermatocytes in third-instar larval testes. The ER in these cells exhibits a striking distribution in two large crescents centred around each spindle pole during metaphase (figure 2*a*), forming domains previously termed astral membranes due to their proximity to astral MTs [20]. Consistent with this terminology, we show that the ER does align very closely with astral MTs (figure 2*a*, arrows), suggesting that interaction with MTs, in addition to centrosomes, may be responsible for the segregation of the ER to spindle poles, similar to what we found in NBs. To determine the dynamics of ER pole segregation, we imaged GFP-Sec61*α* (ER marker) live throughout meiosis. Histone 2A-mRFP (H2A-mRFP) was also imaged to accurately track cell-cycle progression. Similar to NBs, the ER around the NE intensified during prophase as compared with interphase (figure 2*b*; electronic supplementary material, video S4), indicative of ER envelope formation. At the time of NEB (determined by the sudden loss of background H2A-mRFP fluorescence in the nucleus), the ER began to organize on opposite sides of the ER envelope. By metaphase, all the ER appeared to be localized either to the spindle poles or the ER envelope layers, suggesting dramatic rearrangement from its interphase distribution. Importantly, the spindle pole domains persisted throughout anaphase and telophase such that each domain was clearly partitioned separately into each of the two progeny cells. Thus, like in NBs, a significant proportion of the ER is segregated to spindle poles early in cell division due to interaction with centrosomes and their associated astral MTs, and this segregation results in specific partitioning of the organelle to the two progeny cells.

We next tested the specific role of centrosome-dependent astral MTs in spermatocyte ER segregation by analysing *asl* mutants. Live imaging clearly showed disrupted ER positioning and segregation in *asl* mutant spermatocytes. Most notably, the spindle pole enrichment of ER was completely absent in these cells (figure 2*c*, right panels, arrows; electronic supplementary material, video S5). This is consistent with the loss of astral MTs in the mutants, and was in stark contrast to heterozygous control spermatocytes (figure 2*c*, left panels). Importantly, the *asl* mutant spermatocytes exhibited ER structures that were distant from the spindle poles and that were never seen in controls (figure 2*c*, arrowheads; electronic supplementary material, video S5). This suggests that loss of ER at spindle poles due to loss of astral MTs in *asl* mutants leads to major ER positioning and partitioning defects. Our fixed analysis confirms the lack of astral MTs and mislocalized ER (figure 2*d*). However, this analysis revealed an additional finding—although mislocalized away from the spindle poles, ER accumulations in mutants remain localized with distant clusters of MTs. This strongly supports a model in which the ER remains tightly linked with MTs during mitosis/meiosis, where previous studies have mainly focused on this association during interphase. To explore this further, we also analysed *abnormal spindles* (*asp*) mutant spermatocytes. Asp is required for proper spindle formation through a poorly understood MT cross-linking function [21–23]. Our fixed analysis revealed that ER organization was also severely disrupted in *asp* mutant spermatocytes (figure 2*e*). Notably, instead of being organized around spindle poles as in heterozygous controls, the ER in mutants was associated with abnormal MT structures displaced away from the spindle apparatus (figure 2*e*, arrowheads). Thus, as in *asl* mutants, the ER appeared to remain linked with MTs despite the MTs themselves being abnormally organized. These collective results from spermatocytes clearly show a strong association of the ER with MTs during meiotic divisions, and these associations occur specifically with astral MTs when spindles are formed normally.

**Figure 3.**
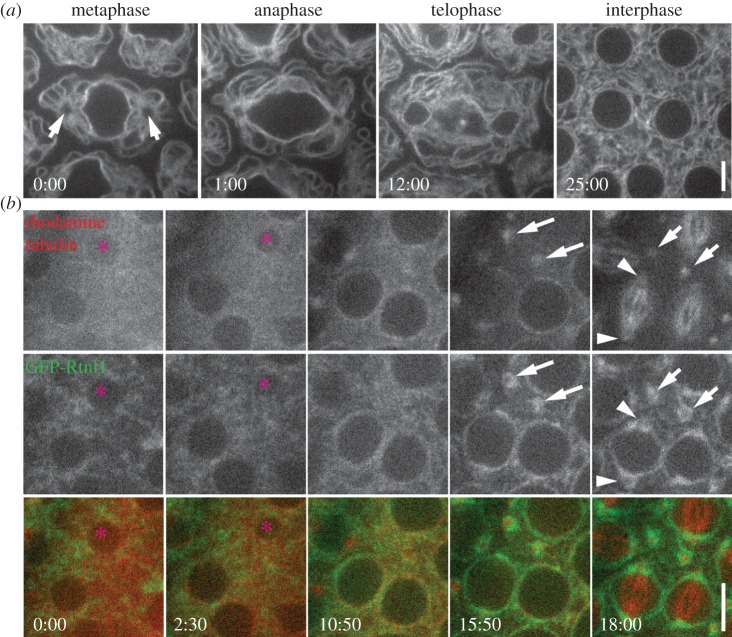
The ER is organized by centrosomes during embryonic nuclear divisions. (*a*) A stage 9 embryo expressing GFP-Sec61*α* was imaged live through the course of a single mitotic division. The location of the two centrosomes (arrows) is clear in the metaphase image; note the radial organization of the ER around the centrosomes. (*b*) A Rtnl1-GFP (green) expressing embryo was injected with rhodamine-labelled *α*-tubulin (red). Fallout of a single nucleus (pink asterisk) can be seen between the 0:00 and 10:50 timepoints. As the syncytium begins the next mitosis (15:50 timepoint), two free centrosomes that organize ER membranes can be detected (arrows). These free centrosomes maintain ER association during metaphase (18:00 timepoint), when other centrosomes still associated with nuclei have formed spindles (arrowheads). Times, min:s; scale bar, 5 µm.

We next investigated ER distribution and segregation behaviour during the syncytial nuclear divisions of the *Drosophila* embryo. Similar to NBs and spermatocytes, we observed robust spindle pole segregation of the ER. During metaphase, the ER displayed a striking radial organization around the centrosome at each pole of a dividing nuclear unit. This pole-associated organization persisted through anaphase and into telophase, following which the ER dispersed throughout the interphase syncytium (figure 3*a*; electronic supplementary material, video S6). Previous analyses have described the spindle pole segregation of the ER in *Drosophila* embryos, but failed to document the radial pattern around the centrosomes that is almost certainly astral MT-dependent [6]. This advance over previous studies is probably due to improvements in imaging technology. We were unable to directly test the role of centrosomes in organizing the ER in the embryo as acentrosomal embryos are not viable [24]. Instead, we took advantage of a unique situation resulting from ‘nuclear fallout’ events in which damaged nuclei dissociate from centrosomes and are reabsorbed into the centre of the embryo, leaving behind free centrosomes near the embryo cortex [25]. These free centrosomes can still nucleate MT asters in-phase with the embryonic nuclear cycle [26]. By imaging the ER (GFP-Rtnl1) and MTs in live embryos, we reliably identified nuclear fallout events in wild-type embryos (figure 3*b*, asterisk) that produced a pair of free, cortically anchored MT asters (figure 3*b*, arrows). As the embryo entered the next mitotic cycle, these two remnant asters clearly recruited ER membranes (figure 3*b*, arrows). This suggests that centrosomes with their associated astral MTs can autonomously recruit ER membranes, without contributions from other spindle or nuclear components.

### 3.3. Endoplasmic reticulum partitioning by spindle pole microtubules is conserved across species

**Figure 4.**
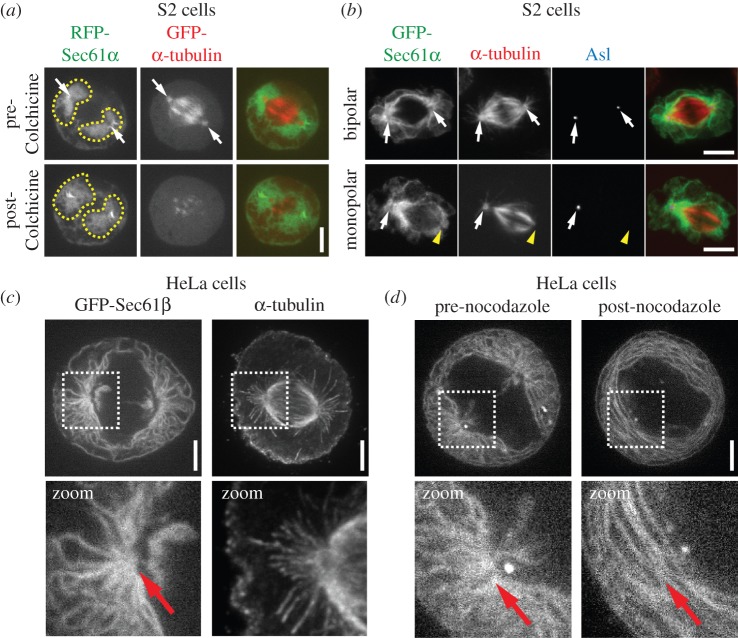
The ER is organized by spindle poles and astral MTs in human and *Drosophila* tissue culture cells. (*a*) A metaphase *Drosophila* S2 cell expressing RFP-Sec61*α* (green) and GFP-*α*-tubulin (red) was imaged live pre- and post-treatment with 100 µM colchicine. The post-colchicine images were taken 20 min following application of the drug. Spindle poles (arrows) and the ER domains surrounding the spindle poles (dotted lines) are indicated. (*b*) GFP-Sec61*α* (green) expressing S2 cells were fixed and stained for *α*-tubulin (red) and Asl (blue). The top panel shows a normal metaphase cell with two centrosomes, marked by Asl (arrows) at both spindle poles. The bottom panel shows a metaphase cell with only a single centrosome located at one of the spindle poles (arrow); the other spindle pole is acentrosomal (yellow arrow head). Note that the acentrosomal spindle pole completely lacks astral MTs and is associated with much less ER than the centrosome-containing pole. (*c*) Shown on the left is a live metaphase HeLa cell expressing GFP-Sec61β, and shown on the right is a fixed metaphase HeLa cell stained for *α*-tubulin to show the organization of a typical spindle in these cells. Note the radial arrays of ER that are organized around the spindle poles and extend towards the cell cortex, and the resemblance of these arrays to the astral MTs seen in the *α*-tubulin image (highlighted in zoomed images below). (*d*) A metaphase GFP-Sec61β expressing HeLa cell was imaged live pre- and post-treatment with 10 µM nocodazole. The post-treatment image was taken 20 min following application of the drug. A clear loss of the ER radial array can be seen following MT depolymerization (highlighted in zoomed images below). Scale bars, 5 µm.

The consistent astral MT-dependent segregation of the ER to spindle poles we found thus far in intact *Drosophila* tissue cells prompted us to examine the conservation of this mechanism. We turned to cultured mammalian cells; we also characterize ER morphology in cultured *Drosophila* S2 cells to serve as a more direct comparison. We found that the majority of the ER in S2 cells was organized in two discrete clusters around the spindle poles (figure 4*a*), very similar to the situation in intact tissues. MT depolymerization with colchicine caused partial dispersion of these clusters (figure 4*a*; electronic supplementary material, video S7), suggesting that MT associations may not be a strict requirement for maintaining ER localization at spindle poles in these cells. Alternatively, MT–ER associations may be important for the initial segregation of the ER to spindle poles early in mitosis in S2 cells but may not be required to maintain the ER at these sites at later stages when our colchicine treatments were conducted. We therefore performed another experiment by analysing mitotic S2 cells that only contained one functional centrosome, a common occurrence in these cells due to inherent dysregulation of the centrosome cycle [27]. These cells formed mitotic spindles with only one pole containing the centrosome, which also had significantly more associated ER compared with the acentrosomal pole (figure 4*b*). Importantly, the acentrosomal pole completely lacked astral MTs and recruited significantly less ER, consistent with a requirement for astral MTs for spindle pole segregation of the ER.

To address the question of mechanistic conservation, we examined HeLa cells, a cell type in which the ER becomes nearly completely dissociated from MTs during mitosis [9,16], leading to the wide-spread conclusion that segregation of the ER occurs via a MT-independent mechanism in these cells. However, most analyses have focused on inner spindle MTs as opposed to astral MTs. The inner spindle region was in fact nearly completely devoid of ER in metaphase HeLa cells except for two small hubs adjacent to the spindle poles (figure 4*c*). However, in the cortical region of the cell where the ER was most densely situated, the ER exhibited conspicuous radial extensions that appeared to be organized around the spindle poles, very similar to the organization of astral MTs in this area. Consistent with a role for astral MTs in this radial ER distribution, MT depolymerization caused a complete loss of the highly organized astral-like array of the ER at the spindle poles (figure 4*d*). Importantly, following drug treatment, the organelle now exhibited concentric rings that lacked any particular organization or anchoring. Because prior to treatment the ER was only distributed around astral MTs, this result strongly suggests a specific role for astral MTs in the organization and localization of the ER in human cells.

### 3.4. The endoplasmic reticulum envelope membranes are actively segregated by spindle microtubules

**Figure 5.**
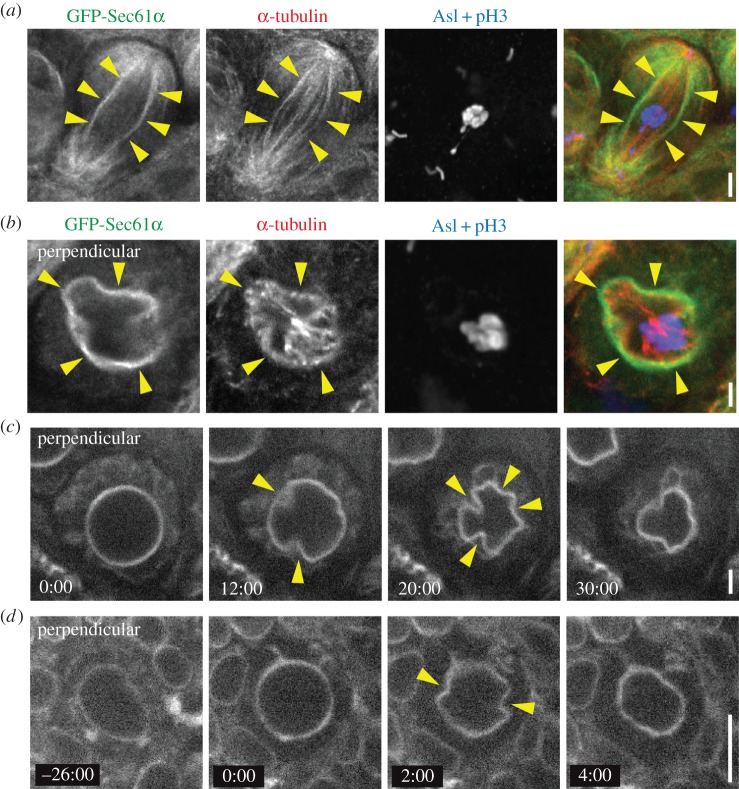
The ER envelope associates with peripheral interpolar spindle MTs. (*a*) A fixed GFP-Sec61α (green) spermatocyte at metaphase of the first meiotic division was immunostained for *α*-tubulin (red), asterless (Asl, blue) to localize centrosomes and phosphorylated histone 3 (pH3, blue). The ER envelope (arrowheads) overlaps with the outermost interpolar MTs of the spindle. (*b*) A metaphase spermatocyte fixed and stained as in (*a*) was imaged perpendicular to the spindle axis. Clear deformations of the ER envelope (arrowheads) are associated with interpolar bundles of MTs. (*c*) A meiotic GFP-Sec61*α* expressing spermatocyte that divided with its spindle perpendicular to the imaging plane was imaged live. Inward deformations of the ER envelope are also seen here (arrowheads). (*d*) A mitotic GFP-Sec61*α* expressing NB was imaged live perpendicular to the spindle axis to show deformations of the ER envelope (arrowheads). Times, min:s; scale bar, 5 µm.

Our results suggest that astral MT association is a common mechanism of mitotic ER positioning in *Drosophila* and human cells. However, a notable difference is the persistence of an ER envelope that surrounds the spindle apparatus throughout cell division in *Drosophila*. The significance of this ER envelope is not known, nor are the mechanisms that regulate its organization. We hypothesized that the ER envelope membranes are linked to spindle MTs, as opposed to astral MTs, thus coupling the entire ER to the spindle apparatus. Consistent with this, the ER envelope aligned perfectly with the outermost bundles of spindle MTs in metaphase spermatocytes (figure 5*a*). It is clear that these MTs do not contact the chromosomes, and thus they can be classified as non-kinetochore or interpolar MTs. Because we imaged spermatocytes in intact, non-dissociated tissue, we were also able to image spermatocytes that divided with their spindles oriented perpendicular to the imaging plane. This revealed that the ER envelope formed a continuous layer that immediately surrounded the outermost ring of spindle MTs (figure 5*b*). Importantly, the ER envelope precisely followed the irregular, non-circular geometry of the outer MT ring, suggesting a physical dependence of the ER envelope membranes on the MTs. Live imaging of spermatocytes dividing in the perpendicular orientation further supported this hypothesis as the ER envelope first achieved a perfectly circular geometry early in meiosis, indicating that these membranes form a complete sphere around the entire spindle (figure 5*c*; electronic supplementary material, video S8). However, the ER envelope next underwent a striking inward deformation that initiated at several discrete points, appearing as if the membranes were being pulled inward at these sites (figure 5*c*, arrows). These deformations resulted in a non-circular geometry similar to that seen in fixed images (figure 5*b*), suggesting that interactions of the ER envelope with outer spindle MTs may drive these deformations. We also observed similar ER envelope deformations in NBs (figure 5*d*) by imaging live, perpendicular spindles. These results indicate that the entire ER network is linked to both astral and the outermost interpolar MTs.

## 4. Discussion

**Figure 6.**
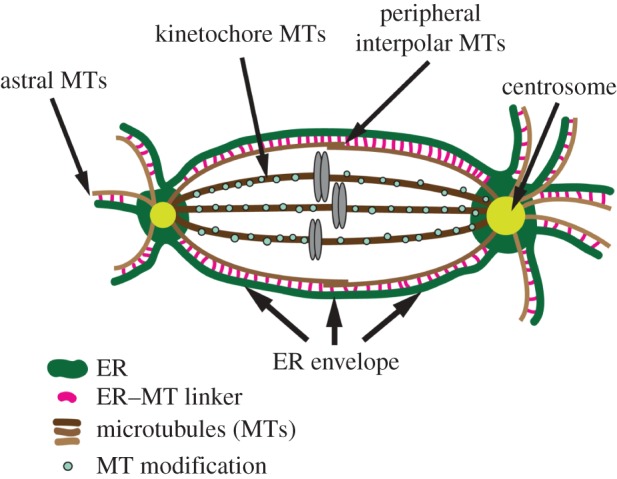
Model depicting the association of the ER (green) with spindle MTs (brown). Centrosomes (yellow) and chromosomes (grey) are indicated. The entire ER network associates with either astral MTs or peripheral interpolar MTs, but not kinetochore MTs. This association is mediated by an ER–MT linker protein (pink), the identity of which is unknown. The exclusion of ER from kinetochore MTs may be due to a MT modification (blue circles) that prevents interaction of the ER–MT linker. This model depicts asymmetric centrosomes, and the amount of associated ER is a direct function of asymmetric astral MT density. This gives rise to asymmetric ER partitioning in NBs. In symmetrically dividing cells like spermatocytes (not depicted), symmetric MT densities around the two centrosomes results in symmetric ER partitioning.

The mechanisms that regulate ER partitioning in dividing animal cells are far from clear, and the fundamental issue of whether the organelle is actively partitioned or stochastically distributed remains controversial. These controversies may be due to several factors in previous studies that have addressed the issue of ER partitioning: first, several recent studies have relied on cultured, transformed cell lines such as HeLa cells that may not recapitulate physiologically relevant processes [9,16,28]; second, potential active partitioning mechanisms, such as spindle MT interactions, have not been directly tested; and third, most analyses have focused on ER interactions with the inner spindle MTs while largely neglecting astral MTs. Our current study addresses these issues by combining genetic and pharmacological manipulations with analysis of ER partitioning *in vivo* in intact *Drosophila* tissues, as well as in cultured cells. Our results show that in the *Drosophila* cell types examined, the ER is recruited to centrosomes early in cell division, probably through interactions with centrosomal MTs. This recruitment in prophase is concomitant with centrosome maturation, the process whereby more pericentriolar material is recruited to the centrosome to afford greater MT nucleation and anchorage in preparation for spindle formation. Several models could explain this increase in recruitment of ER to mature centrosomes and astral MTs. One model is that cells simply use the same mechanism of linking the ER and MTs in both interphase and mitosis. Therefore, by increasing MT density at the centrosome in mitosis, more ER is recruited and concentrated at the developing poles. An alternative model is that a new ER–MT linking mechanism is engaged, or activated specifically in mitosis, potentially through regulation by mitotic cyclin/cdks [15]. There is precedence for controlling the ER–MT linkage in a cell-cycle-specific manner—STIM1 phosphorylation in mitosis disengages the ER from spindle MTs [16]. Although this is a form of negative regulation, it is not unreasonable to hypothesize the presence of a parallel positive regulatory mechanism. In either model, it is still puzzling as to why the ER is not recruited to all MTs—why is it specific to astral and peripheral interpolar MTs? One hypothesis is that the MT density, or the viscosity of the spindle, forms a physical barrier that prevents ER entry to the interpolar region. We do not favour this hypothesis because we know that artificially linking the ER to MTs using a non-phosphorylatable STIM1 construct can force the ER deep into the spindle region [16]. We favour an alternative hypothesis, whereby specific subpopulations of MTs convey ER-binding capability, while others do not. How might this occur? We are beginning to appreciate the complexity of MT modifications, which can have dramatic effects on MT behaviour and function. A very relevant finding is the presence of detyrosinated tubulin exclusively within the spindle and not in astral MTs [29,30]. One plausible hypothesis is that an unknown ER–MT linker protein cannot bind detyrosinated MTs, which would result in ER exclusion from the spindle region (figure 6). This would be an exciting future direction because of its implication in asymmetric stem cell divisions—one might envision that unique MT modifications could exist on the apical versus basal spindle poles. In addition, MT motor-dependent ER sliding events preferentially occur on acetylated MTs, further supporting a role for MT modifications in dynamic ER regulation [31].

As the cell proceeds through subsequent stages of mitosis, the ER remains associated with astral MTs, resulting in active partitioning of the organelle to the two progeny cells. We further show that disruption of centrosomes and astral MTs leads to ER partitioning defects, confirming the obligate role of these cytoskeletal structures. These mechanisms are not limited to *Drosophila* cells, as we show that mitotic ER positioning also depends on astral MTs in human cells. We also present the important and novel finding that the ER is asymmetrically partitioned in asymmetrically dividing neural stem cells. This ER asymmetry is probably dependent on centrosomal and MT asymmetry [17,18], and results in a higher concentration of ER in the regenerating stem cell compared with the differentiating GMC. Thus, our data suggest that association of the ER with astral MTs may be a universal mechanism of ER partitioning that can be adapted within specific cellular or developmental contexts such as during asymmetric stem cell division. Further, our NB results present the provocative possibility that ER partitioning may have a specific role in the mechanism of asymmetric cell division or tissue development.

Identification of the molecular factors that link the ER with spindle MTs is the key to further understanding the role of ER partitioning in organismal physiology and development. Importantly, it is likely that the molecular features of ER partitioning, including the specific factor that links the ER to astral MTs, are highly conserved as we see similarities in systems as disparate as mitotic HeLa and meiotic *Drosophila* spermatocytes. We believe that *Drosophila*, with its powerful genetic tools and *in vivo* analyses, is an ideal system in which to further investigate these mechanisms. A distinct possibility is that MT motors are involved, and systematic analysis of all known *Drosophila* MT motors is a clear and tenable approach. Other proteins such as spastin, Climp-63 and REEPs have also been shown to associate the ER with MTs in various cell types [4], and possible roles of these proteins in spindle MT attachment should be studied further. Most significant among these, it was recently shown that REEP3 and REEP4 are required for spindle pole focusing in HeLa cells, suggesting a potential role for these proteins in mitotic ER partitioning [32,33].

In conclusion, our results demonstrate that interaction of the ER with spindle MTs is a conserved mechanism that controls the distribution of the organelle during animal cell division. This facilitates equal partitioning of the organelle during symmetric cell divisions, but also allows for specific regulation of ER distribution during specialized processes such as asymmetric cell division. *Drosophila* will be a powerful system moving forward to identify the specific molecular mechanisms involved and the functional roles of ER partitioning in organismal physiology.
